# A Physics-Informed Convolutional Neural Network with Custom Loss Functions for Porosity Prediction in Laser Metal Deposition

**DOI:** 10.3390/s22020494

**Published:** 2022-01-10

**Authors:** Erin McGowan, Vidita Gawade, Weihong (Grace) Guo

**Affiliations:** 1 Department of Mathematics, Rutgers University, New Brunswick, Piscataway, NJ 08854, USA; egm68@scarletmail.rutgers.edu; 2 Department of Industrial and Systems Engineering, Rutgers University, New Brunswick, Piscataway, NJ 08854, USA; vag65@soe.rutgers.edu

**Keywords:** laser-based additive manufacturing, deep learning, in situ porosity detection, data fusion, inspection and quality control, sensing, monitoring and diagnostics

## Abstract

Physics-informed machine learning is emerging through vast methodologies and in various applications. This paper discovers physics-based custom loss functions as an implementable solution to additive manufacturing (AM). Specifically, laser metal deposition (LMD) is an AM process where a laser beam melts deposited powder, and the dissolved particles fuse to produce metal components. Porosity, or small cavities that form in this printed structure, is generally considered one of the most destructive defects in metal AM. Traditionally, computer tomography scans measure porosity. While this is useful for understanding the nature of pore formation and its characteristics, purely physics-driven models lack real-time prediction ability. Meanwhile, a purely deep learning approach to porosity prediction leaves valuable physics knowledge behind. In this paper, a hybrid model that uses both empirical and simulated LMD data is created to show how various physics-informed loss functions impact the accuracy, precision, and recall of a baseline deep learning model for porosity prediction. In particular, some versions of the physics-informed model can improve the precision of the baseline deep learning-only model (albeit at the expense of overall accuracy).

## 1. Introduction

### 1.1. Background of Laser Metal Deposition and Challenges

Laser metal deposition (LMD) is an additive manufacturing (AM) process that uses a laser beam to melt powder as it is deposited and fuses those dissolved particles to build metal components. LMD is capable of producing a functional part directly from a three-dimensional (3D) computer aided design model, and is able to do so using more than one material simultaneously [1]. LMD has the advantages of a very high material build-up rate, 3D surface adaptability, and gradient layers. It can reduce the waste created during production, and it suits the production and repair of comprehensive and customized parts [2]. In one successful case, LMD printed a helicopter engine combustion chamber and achieved wall density of more than 99.5% throughout the part [3]. For these reasons, LMD is used in the production of high value-added parts in the aerospace, automotive, energy, petrochemical, and biomedical industries [4].

The existence of porosity, or small cavities that form in this printed structure, is generally considered one of the most significant quality issues in metal AM parts. Pores may form from processing defects (e.g., “lack of fusion” porosity due to insufficient energy input and “keyhole” porosity that occurs due to overheating) or raw material defects (e.g., “trapped gas” porosity inherited from the powder feedstock and “soluble gas” porosity). These tiny pores can undermine the integrity of the final structure by reducing static mechanical properties and causing significant scatter of fatigue. Various models and techniques have been developed to improve the AM process and to minimize porosity. For example, Oskolkov et al. developed an indirect temperature measurement method to improve mechanical properties of printed materials by maintaining control of extruded material temperature using high-frequency induction [5]. They also developed a regression model to predict the amplitude of the desired signal (dimensionless units) using nozzle temperature and magnetic field strength. Other existing methodologies are focused on tracking general process changes via statistical process control [6], in situ monitoring of melt pool images to understand microstructure formation [7], tracking porosity via thermal distribution [8], or modeling layer-wise spatial distribution [9]. However, process instability is difficult to eliminate, making it impossible to avoid porosity altogether.

### 1.2. Physics Models in LMD

Physics-based models that do not incorporate deep learning strategies can help understand the nature of pore formation and its characteristics. For example, theoretical finite element models using Goldak’s moving heat flux due to a moving heat source are beneficial to simulate thermal history to help form a prediction on the distortion of the process [10]. Goldak’s model can provide insight on thermal processes and how it impacts mechanical and microstructural properties of AM materials. For example, it can benefit the study that accounts for realistic microstructural development on deformation from grain structure evolution by providing insight on thermal processes [11]. However, these models have several limitations. First, the model parameters are difficult to calibrate due to incomplete physics-related data. Moreover, simulated models cannot capture the realistic situations of the uncertainty of the complex process and are infeasible for real-time applications.

The AM process is complex, as when the deposit is melted and becomes liquid, convection flow occurs, causing alloying element vaporization and increased thermal gradients [12]. In LMD, the laser beam melts a stream of material feedstock, forming melt pools which then solidify due to cooling as the laser continues its path. After solidifying, the material goes through more heat cycles as new layers are built on top of previously solidified layers. During melt pool formation, porosity can form and can be monitored using temperature profiles and geometry. However, aspects such as the laser–particle interaction, hydrodynamic fluid flow of the melt pool, and solidification are also important to consider. Indeed, solidification can be impacted by thermal behavior metrics such as cooling rates and temperature gradient pool geometry [13]. The simulation model used in our paper does relate information about the laser–particle interaction, fluid flow, and solidification impacting melt pool geometry during the build that the pyrometer sensor data alone cannot capture.

Apart from thermal behavior, process-induced porosity can occur due to microstructure grain evolution. Two ways to simulate grain evolution are using the phase field method and cellular automaton. The phase field method uses a phase field variable and governing physics equation to describe the state of material as a function of position and time [14]. By incorporating knowledge of the state of the material (liquid or solid) at a specific location, realistic results occur for simple microstructure shapes using simple numerical methods, yet computing time is very long. The cellular automaton uses the physical knowledge of nucleation and grain growth to study grain evolution while solidification occurs in the printing process [15]. Yet, ref. [15] used a weak version of the cellular automaton coupled with finite element modeling by neglecting nucleation influence. This model also neglected solid state phase change and recrystallization. While the cellular automaton is computationally more efficient, the phase field method captures finer information about the grain structure.

Other works showed qualitative [16] and quantitative [17] acceptance between simulated and experimental outputs by combining a 3D cellular automaton with finite volume to predict grain structure. In all cases of the finite element, finite differences, phase fields, and cellular automaton models, computation time seems to be long if the goal is fine accuracy, while assumptions, simplicity, and negligence of the process result in faster results but lower accuracy.

### 1.3. In Situ Monitoring Techniques in LMD

A review of in situ monitoring and metrology found that camera-based sensors that monitor the powder bed condition, geometrical accuracy, and build height, and sensors such as pyrometry and IR cameras that monitor temperature, are limited for in situ closed-loop process control due to existing licenses and harsh environments. In situ methods only show information about the component surface, and are not yet able to identify material porosity from surface topography [18]. There is a tradeoff between spatial capability (increased information) and time penalty (increased scan time results in longer production time) to understand one aspect of integrating non-destructive evaluations methods for in situ monitoring in AM [19].

These in situ monitoring techniques are integral to understanding the microstructural development and are used to create models that predict the process-induced porosity. In [20], the material ratio curves were studied to differentiate AM topographies and correlate volume with open surface pores. Currently, the in situ monitoring techniques help relay information to the models but do not identify porosity in real time. Instead, the models are currently being studied to identify the porosity with some accuracy, and the next step is to allow the in situ monitoring sensors to relay information to the printing system to perform real-time fixes.

The LMD process, like other AM processes, has the potential to equip multiple sensors including thermocouples, infrared temperature sensors, and accelerometers. While sensors are powerfully accurate, cheap, and can provide real-time insight into the printing process, they can be prone to errors. With that, physics-based data can be combined with sensors through various methods. One can use simple domain knowledge that the temperature observed by a sensor should always be positive, for example, and if it were not, the custom loss function would penalize that incorrect sensor reading. In our work, we combine the pyrometer-based sensor data that dynamically observe the thermal behavior with numerical approximations of partial differential equations (PDEs) that statically predict thermal behavior of the process to help predict the process-induced porosity and negate any inaccurate sensor readings. A proton transfer reaction time-of-flight mass spectrometry sensor monitored in real-time the concentration of compounds and helped to build a classification model to predict if concentrations exceeded a limiting value [21]. However, ref. [21] admits it falls short in understanding the long-term effects of the sensor’s lifetime since it studied the sensor only for five weeks (i.e., sensor drift can occur, and re-occurrence of electrochemical sensors can be affected in the long run).

Hence, incorporation of physics-based knowledge can improve a model’s predictive performance by serving as a check on what sensors collect in the case said sensors collect inaccurate measurements. Many works show sensory applications to the incorporation of a physics-informed machine learning (PIML) model. One challenge of integrating PIML into multimodal sensor data is matching modalities of different sensors. In one way, Guo et al. appended finite element analysis (FEA)-simulated data to a convolutional neural network (CNN) model that incorporated thermal images from sensors [22]. In another way, Gawade et al. matched the FEA-simulated melt pool data to thermal melt pool images by similar location and time [23]. This paper incorporates FEA data into the custom loss functions of the CNN model, which takes the pyrometer sensor-based data as its baseline model data.

### 1.4. Machine Learning-Based Models in LMD

To deal with such uncertainties, machine learning and deep learning models that utilize the sensor data collected during the real manufacturing processes can be developed. These models can efficiently handle complex data (with high dimensionality, heterogeneity, large volume), and can be potentially used to predict porosity accurately during the LMD process. For example, Khanzadeh et al. developed a porosity prediction method based on the temperature distribution of the top surface of the melt pool by using self-organizing map clustering [8]. Khanzadeh et al. used morphological characteristics of the melt pool boundary to investigate the relationship between the melt pool characteristics and the defect occurrence in an as-built AM part [24]. Tian et al. developed a deep learning-based data fusion method for in situ porosity detection in laser-based AM [22]. Yang et al. developed a CNN model to investigate how the melt pool can be characterized in real time for feedback control of a laser powder bed fusion AM process [25]. However, these purely deep learning models use black-box methods that do not incorporate valuable physics knowledge. They also must be carefully trained with available experimental data, and often require a lot of data to be trained upon. Such models are difficult to interpret, apply, or generalize for a broader set of process conditions [26].

### 1.5. PIML Models in AM

Recently, an emerging research area that integrates the physics-informed (or physics-based, physics-driven, physics-guided) models and machine learning models is gaining increased interest [26,27,28,29,30]. These methods combine the strengths of machine learning and physical principles to decrease uncertainty from PDEs, data (noise), and learning models themselves (generalizing error) [27]. Examples of physics-guided ML models used in engineering and environmental systems are uncertainty quantification, inverse modeling, parameterization, and forward solving PDEs [28]. It has also been shown that physical data or constraining the loss function based on physical constraints helps to construct effective physics–deep learning hybrid models [29,31]. Deep learning models seek to minimize loss functions for a given input to evaluate the discrepancy between the true and predicted label output. Thus, a physics-based model and data-driven model are well suited to enhance each other, forming a PIML model.

In the context of AM in particular, PIML models can be developed to study several problems associated with process and product quality, such as melt pool characteristics prediction and porosity analysis. For example, Zhu et al. developed physics-informed neural networks to predict temperature and melt pool fluid dynamics for metal AM [32]. Liu et al. developed a PIML model to analyze the porosity in laser powder bed fusion AM [30]. Recent research has also shown that incorporating physical data into the machine learning model can enhance porosity-predicting models for LMD processes. For example, Guo et al. developed a physics-driven deep learning model for the process–porosity causal relationship and porosity prediction with interpretability [26]. Gawade et al. developed simulated and empirical data-driven insight into supervised learning for porosity prediction [23]. In these works [23,26], the physics-informed insights are integrated into the features that are used in the machine learning models.

These models, however, do not integrate said physics-informed insights into the architecture of the machine learning model itself. This paper is centered on the weakly explored notion of incorporating physics-informed insights into the architecture of a deep learning model via the loss function. The design of the loss function is anotherimportant component in the design of machine learning models [33,34]. Different from previous the works [23,26], this paper focuses on the capability to combine simulated physical data with experimental data in the form of physics-informed custom loss functions built into deep learning models in the LMD process. This paper aims to explore the impact of several physics-informed loss functions on a baseline deep learning-only porosity prediction model.

The rest of the paper is organized as follows: Section 2 explains the empirical and simulated data acquisition process, Section 3 details the method used to construct a baseline deep learning-only model and physics-informed custom loss functions, and Section 4 explores the results and discussion, including a breakdown of training, validation, and testing data set splits, data pre-processing and augmentation, and model performance. Section 5 ties these together, in conclusion, highlighting key outcomes.

## 2. Data Description

Both simulated and experimental data of the melt pool temperature are used in this study. As mentioned earlier, these two categories of data can complement each other. While the sensor data from the experiment can capture the environmental factors and uncertainties, the simulation models can capture more information about the complex printing process. However, the sensors themselves can be prone to systematic and random errors, and the sensors’ accuracy depends on the sensors’ calibration and measurement level. Therefore, it is also important to capture the information from physics-based simulation to negate errors from sensor information.

### 2.1. Experimental Data via Pyrometer

**Figure 1:**
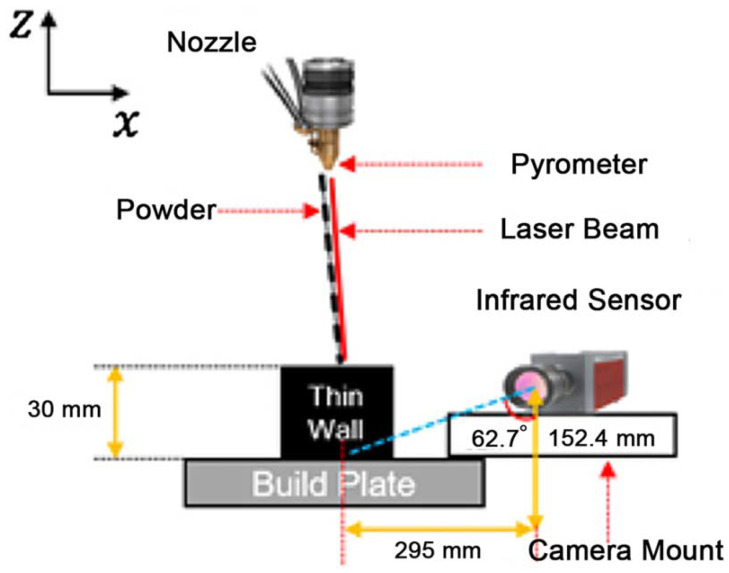
Setup of the pyrometer [35].

In this study, we use the melt pool images taken during the LMD process for printing 60-layer Ti-6Al-4V thin-walled structures, as is used in [35]. These data are measured by a dual-wavelength pyrometer sensor, which is a part of the OPTOMEC Laser Engineered Net Shaping (LENS) 750 printer. The setup of the system including the pyrometer is shown in Figure 1. The pyrometer is mounted above the build plate, outside the chamber, and it monitors the melt pool temperature in a vertical direction. The data of the pyrometer are output to comma separated value (CSV) files, each of which contains a 752 × 480 (width × height) matrix of temperature values. In total, 1564 melt pool images are captured by the pyrometer.

After the part is printed, X-ray computer tomography (CT) is used to catch its interior features, specifically the melt pool porosity, in a non-destructive manner [8]. The measured porosity is used to determine the part quality. The X-ray CT system used was Skyscan 1172 with a 1μm resolution. However, the magnification in this case was a pixel size set to 3 μm. It was attached vertically on a stage and rotated a full 360 degrees. The X-ray beams transferred 100 KV voltage and 100 μ current. For further parameter settings and the software package used for the CT scan and image reconstruction, please refer to Experimental Setup section of [6]. The micro-CT can detect pores at least 0.05 mm in diameter and so pores of diameters ranging from 0.05 mm to 0.99 mm were identified [8].

The CT scan returns an output of the pores as a circumscribed spherical shape with approximate diameter sizes and their locations. Precisely, the CT scan captured pores with an approximate diameter of 0.05 mm or greater at specific locations in the printed part. The pyrometer captures melt pool images in the printed part at specific locations. If a specific location (in a part containing a pore with an approximate diameter greater than 0.05 mm) captured from a CT scan matches a specific location (in part of a melt pool image) captured from the pyrometer, this is an indication that a pore is formed at this location, and then that melt pool image is labeled as a true “bad” image (containing a pore). The CT scan cannot identify pores with an approximate diameter less than 0.05 mm. As a result, the CT scan does not capture a location with pores with an approximate diameter less than 0.05 mm. In that case, any remaining melt pool images are labeled as true “good” melt pool images. This is because any remaining melt pool images’ locations captured by the pyrometer do not match the locations captured from the CT scan, meaning that no pores are formed at those locations. In sum, a melt pool image with the “bad” label indicates that there is a pore of size 0.05–0.99 mm at the location where the melt pool is captured; a melt pool image with the “good” label indicates that there is no pore or a pore of size less than 0.05 mm at the location where the melt pool is captured. Note that, if there is a pore of less than 0.05 mm at a location, the melt pool captured at that location is labeled as “good”. If the prediction for that melt pool image is also “good”, then it is a correct classification; if the prediction is “bad”, then it is a false positive.

### 2.2. Physics-Based Data via FEA

FEA is used to simulate the heat behavior of the process and predict print characteristics in the simulated environment. FEA uses numerical approximations to solve analytical laws of physics that are both space and time dependent. During a print, conduction within the part and convection and radiation between the part and environment release thermal energy. Those thermal gradients can impact stresses and eventual distortion of the print. The moving heat source model within the FEA simulation captures the thermal gradients. In particular, the simulation meshes, activates, and deactivates elements at individual time steps to simulate the thermal behavior of the printing process [36]. The experimental process parameters are used in the simulation. The detailed problem statement and boundary conditions can be found in [26].

The simulated data captures the three-dimensional thermal behavior of the printing process that the pyrometer sensor (limited to information from two-dimensional images) cannot. The FEA model helps provide thermal behavior of the melt pools from numerical approximations using physics laws of the printing process. If there are any data collection errors from sensors or processing, the FEA model data incorporated into the custom physics losses will help provide more information to the model of the printing process’ temperature, and as a result, process-induced porosity. However, the simulation assumes constant gas levels in printers, and thus it cannot capture the actual thermal behavior impacted by events in the chamber (e.g., constantly changing oxygen and argon gas levels, hydrodynamic flow influence, and laser–particle interaction, as well as coupled heat transfer) that can affect the thermal behavior of the process [36]. Nevertheless, the simulation provides a proxy of how the flow influence, coupled heat transfer, and laser–particle interaction will affect the complex melting and solidification process [12]. It is integral to add these numerical approximations of the FEA into the experimental thermography to understand the complex AM process.

Finally, an algorithm augmented simulated and experimental data to have higher predictive power. Precisely, the simulated melt pools were matched to experimental melt pools based on sharing a similar time and location of print. How the FEA model’s melt pools incorporated porosity is explained in [23].

## 3. Method

**Figure 2:**
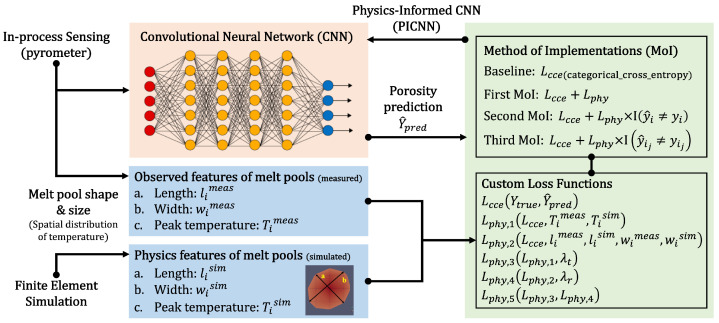
A data fusion for MoI framework that uses matched data from experimental melt pool images with labeled porosity and simulated melt pools to train deep learning models with physics-informed custom loss functions using three different MoIs.

The overall framework of the proposed method is shown in Figure 2. Its top left portion (in-process pyrometer sensing) and bottom left portion (finite element simulation) are explained in Section 2.1 and Section 2.2 , respectively. After the data pre-processing steps described in Section 2, several deep learning models with various methods of implementation (MoIs), as shown in the top right portion of Figure 2, with various custom loss functions are developed, as shown in bottom right portion of Figure 2. First, a deep learning-only model without any physics-informed custom loss function is trained with only the standard categorical cross-entropy loss function $L_{cee}$ (which will be explained in Section 3.2) and used as a baseline. The baseline is compared to 3 different MoIs that help to train the physics-informed convolutional neural network (PICNN) model with the first custom loss function (i.e., $L_{phy,1}$). In the first MoI, the custom loss function is used for all labeled images in the training set, while in the second MoI, the custom loss function is used for only the incorrectly labeled images ($y_{i}$ is a true label of image *i*, and i=1,..,n, where *n* is the number of incorrectly labeled images), while in the third MoI, the custom loss function is used for incorrectly labeled “bad” images only ($y_{i_{j}}$ is a true label of true “bad” image $i_{j}$, and $i_{j}=$$1,..,n_{b}$, where $n_{b}$ is the number of incorrectly labeled true “bad” images). The best MoI is selected based on the validation loss and other performance metrics. Finally, in Figure 2, the best MoI is used to train the PICNNs with 5 different custom loss functions (i.e., $L_{phy,1},\;...,\;L_{phy,5}$), and the impact of custom loss functions on the performance of the models is investigated. Through all of Figure 2, the training performance is evaluated by comparing the predicted porosity labels with true porosity labels.

Next, we introduce the baseline deep learning-only model, the five custom loss functions, and the three MoIs used in this work.

### 3.1. Base CNN

First, a baseline deep learning-only model is constructed based on a CNN. CNNs can effectively reduce many parameters without compromising quality and are well suited to the given image classification task, where the images are high dimensional data. This baseline model uses the same PyroNet model as [22]. In particular, a VGG16 architecture [37] is chosen as the foundation of this model for its small 3 × 3 convolution cores, which increase the depth of the network and allow it to identify more complex patterns. Moreover, the VGG16 architecture has a relatively small number of parameters, a characteristic that decreases computational time.

**Figure 3:**
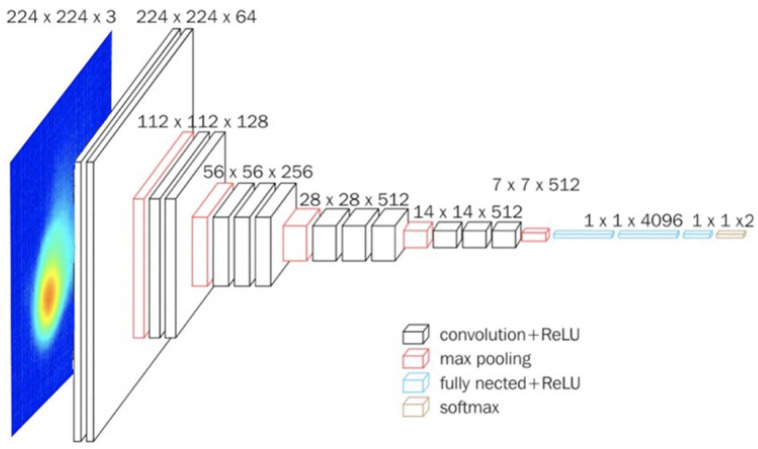
The VGG16 architecture modified for the PyroNet [22].

The CNN model accepts RGB images of pyrometer melt pools with a resolution of 224 × 224, “good” or “bad”, for each image. The VGG16 model (Figure 3) architecture contains 13 convolutional layers (separated into 2 groups of 2 followed by 3 groups of 3), which extract features from the input images and have trainable weights. The kernel size, stride, and pad of all convolutional layers are 3, 1, and 1, respectively. The model also contains 5 max-pooling layers (1 after each group of convolutional layers), which create a feature map of the most prominent features by selecting the maximum element of a given region. The kernel size, stride, and pad of the max pooling layers are 2, 2, and 0, respectively. These are followed by a flatten layer, which transforms the data matrix into a one-dimensional array given to the fully connected layers. Next, two 4096-channel fully connected layers create a one-dimensional feature vector. These are fully connected, followed by a dropout layer with a dropout rate of 0.5 (which removes some of the parameters) to prevent overfitting. Aside from the max pooling and dropout layers, all of the layers above use a rectified linear unit (ReLU) activation function). Finally, a third fully connected layer maps the results of the model up until that point to an interpretable classification label (in this case, “good” or “bad”). This final fully connected layer uses a softmax activation function. Figure S1 shows a summary of the hyperparameters used in this model and in the PyroNet model.

### 3.2. Physics-Informed Custom Loss Functions

This work explores the impact of incorporating five physics-informed loss functions into the baseline deep learning model. Let $L_{phy,i}$ (i=1,...,5) be the $i^{th}$ loss function and *N* be the number of images. The five loss functions are represented in Equations (1) to (5), respectively.

(1) $$L_{phy,1}=L_{cce}\left(y_{true},y_{pred}\right)+\frac{1}{N}\sum_{i=1}^{N}ReLU\left(R_{i}\right)$$

(2) $$L_{phy,2}=L_{cce}\left(y_{true},y_{pred}\right)+\frac{1}{N}\sum_{i=1}^{N}\left[ReLU\left(\frac{l_{i}}{w_{i}}-\left(1+\delta \right)\right)+ReLU\left(\left(1-\delta \right)-\frac{l_{i}}{w_{i}}\right)\right]$$

(3) $$\begin{array}{c} L_{phy,3}=L_{cce}\left(y_{true},y_{pred}\right)+\lambda_{t}\times \frac{1}{N}\sum_{i=1}^{N}ReLU\left({\hat{\epsilon }}_{t_{i}}-\begin{array}{c} \frac{\delta_{t}-\mu_{t}}{\sigma_{t}} \end{array}\right)\\ =L_{cce}\left(y_{true},y_{pred}\right)+\lambda_{t}\times \frac{1}{N}\sum_{i=1}^{N}ReLU\left(\frac{\epsilon_{t_{i}}-\delta_{t}}{\sigma_{t}}\right) \end{array}$$

(4) $$L_{phy,4}=L_{cce}\left(y_{true},y_{pred}\right)+\lambda_{r}\times \frac{1}{N}\sum_{i=1}^{N}ReLU\left(\frac{\epsilon_{r_{i}}-\delta_{r}}{\sigma_{r}}\right)$$

(5) $$L_{phy,5}=L_{cce}\left(y_{true},y_{pred}\right)+\lambda_{t}\times \frac{1}{N}\sum_{i=1}^{N}ReLU\left(\frac{\epsilon_{t_{i}}-\delta_{t}}{\sigma_{t}}\right)+\lambda_{r}\times \frac{1}{N}\sum_{i=1}^{N}\left(\frac{\epsilon_{r_{i}}-\delta_{r}}{\sigma_{r}}\right)$$

where $L_{cce}\left(y_{true},y_{pred}\right)=-\sum_{i=1}^{C=2}t_{i}log\left(s_{i}\right)=-t_{1}log\left(s_{1}\right)-\left(1-t_{1}\right)log\left(1-s_{1}\right)$ denotes the loss calculated by the standard categorical cross-entropy loss function, which is part of the Keras library. On its own, this categorical cross-entropy loss function measures the discrepancy between the true (or actual) label for each image ($y_{true}$) and the label assigned to each image by the model (predicted image or $y_{pred}$). The categorical cross-entropy loss accounts for *C*, the number of classes the images are being sorted into (e.g., “good porosity” and “bad porosity”), $t_{i}$ is a tensor containing the true labels for all of the images in the batch, and $s_{i}$ is a tensor containing the predicted labels for all of the images in the batch. In our case, it follows that $t_{1}$ and $s_{1}$ are tensors containing the true labels and predicted labels for Class 1, respectively. Moreover, $\left(1-t_{1}\right)$ and $\left(1-s_{1}\right)$ are tensors containing the true labels and predicted labels for Class 2, respectively. To be clear, the usage of “class” here refers to the general categories of “good porosity” and “bad porosity” that images may be sorted into, while the usage of “label” refers to the specific class assigned to a single given image.

In $L_{phy,1}$, $R_{i}:=T_{i}^{meas}-T_{i}^{sim}$ is the residual maximum melt pool temperature (i.e., the difference between the measured (empirical) maximum melt pool temperature $T_{i}^{meas}$ and the simulated maximum melt pool temperature $T_{i}^{sim}$) for image *i*. As mentioned above, “keyhole” porosity is a type of porosity defect that occurs due to the melt pool overheating during the LMD process. To target and identify instances of this type of porosity, $L_{phy,1}$ penalizes instances where the observed (empirical) maximum melt pool temperature is greater than the simulated (ideal) maximum melt pool temperature (where the usage of the term “penalty” refers to an increase in loss). Expressly, residual maximum melt pool temperature $R_{i}$ of each image *i* creates a tensor. Subsequently, a rectified linear unit (ReLU) activation function removes any negative values of $R_{i}$, leaving only the values corresponding to images where the melt pool overheating occurs. The mean of these values is then added to the loss function.

In $L_{phy,2}$, $l_{i}$ and $w_{i}$ are the empirical melt pool length and width for image *i*, respectively. Furthermore, $L_{phy,2}$ penalizes any instance in which the empirical length-to-width ratio of a given melt pool deviates from the (ideal) simulated length-to-width ratio by more than δ. As the simulated length-to-width ratio is 1 for every melt pool, this simply means that any empirical length-to-width ratio between 1−δ and 1+δ is filtered out by the ReLU activation function after the calculations $\frac{l_{i}}{w_{i}}-\left(1+\delta \right)$ and $\left(1-\delta \right)-\frac{l_{i}}{w_{i}}$. When the empirical melt pool length-to-width ratio falls outside of these bounds, the amount that it exceeds the corresponding bounds (e.g., if the empirical melt pool length-to-width ratio for a given image is 1.2 and δ=0.1, then the entry for that image in the tensor would be 0.1) leaves a tensor of values. The mean of these values is added to the loss function. The simulated length-to-width ratio provided by the FEA model is 1 for every melt pool from the data source which is a current limitation provided by the simulated data source. The simulated model’s data can be improved to capture more realistic situations of the differing length and width for a given melt pool as they may not always be the same. In experiments, the length-to-width ratio will differ from 1 for each melt pool. This ratio can be manipulated to adjust to true situations for future study. The idea behind tracking the ratio is that there may be a specific ratio (resulting from an odd-sized melt pool from either a too-large width, small length, or vice versa) that might result in an unhealthy melt pool that could cause a pore to develop.

In $L_{phy,3}$, $\lambda_{t}$ is the coefficient scaling temperature-informed term, and $\epsilon_{t_{i}}$ and ${\hat{\epsilon }}_{t_{i}}$ are the relative errors between the empirical maximum melt pool temperature and the simulated maximum melt pool temperature for image *i* before and after normalization, respectively. Moreover, $L_{phy,3}$ penalizes any instance in which the relative error $\epsilon_{t_{i}}$ exceeds a threshold $\delta_{t}$ (before normalization). The relative error before normalization, $\epsilon_{t_{i}}$, is calculated as $\epsilon_{t_{i}}=\left|R_{i}/T_{i}^{sim}\right|$. Then, the normalized percent error for each melt pool image ${\hat{\epsilon }}_{t_{i}}$ can be calculated as ${\hat{\epsilon }}_{t_{i}}=\left(\epsilon_{t_{i}}-\mu_{t}\right)/\sigma_{t}$, where $\mu_{t}$ and $\sigma_{t}$ are the average and standard deviation of the percent errors for all images, respectively. Correspondingly, a ReLU activation function that filters out any instances in which the normalized percent error does not exceed $\delta_{t}$ can be created, as shown in Equation (3).

In $L_{phy,4}$, $\lambda_{r}$ is the coefficient scaling length-to-width ratio-informed term, and $\epsilon_{r_{i}}$ and ${\hat{\epsilon }}_{r_{i}}$ are the relative errors between the empirical melt pool length-to-width ratio and the simulated melt pool length-to-width ratio for image *i* before and after normalization, respectively. Moreover, $L_{phy,4}$ penalizes any instance in which the relative error $\epsilon_{r_{i}}$ exceeds a threshold $\delta_{r}$ (before normalization). The relative error before normalization, $\epsilon_{r_{i}}$, is calculated as $\epsilon_{r_{i}}=\left|\left(\frac{l_{i}}{w_{i}}\right)-1\right|$, as the simulated melt pool length-to-width ratio is always 1. Then, the normalized percent error for each melt pool image can be calculated as ${\hat{\epsilon }}_{r_{i}}=\frac{\left(\epsilon_{r_{i}}-\mu_{r}\right)}{\sigma_{r}}$, where $\mu_{r}$ and $\sigma_{r}$ are the average and standard deviation of the percent errors for all images, respectively. Correspondingly, a ReLU activation function filters out any instances in which the normalized percent error does not exceed $\delta_{r}$, as is shown in (4). Please note that the principal difference between $L_{phy,3}$ and $L_{phy,4}$ is that the former penalizes instances in which the maximum melt pool temperature exceeds the threshold, while the latter penalizes instances in which the melt pool length-to-width ratio exceeds the threshold.

Finally, $L_{phy,5}$ combines $L_{phy,3}$ and $L_{phy,4}$ to take both the simulated and empirical maximum melt pool temperature as well as the simulated and empirical measures of melt pool length and width into account.

In this study, the threshold values in Equations (2) to (5) are set as $\delta =\delta_{t}=\delta_{r}=0.1$.

### 3.3. Methods of Implementation (MoIs)

After the physics-informed custom loss functions are defined, they are implemented by using three different MoIs.

The first MoI replaces the standard categorical cross-entropy function used for the baseline deep learning-only model (i.e., $L_{cce}\left(y_{true},y_{pred}\right)$) with one of the physics-informed custom loss functions described above in Equations (1) to (5). In other words, the first MoI adds physical data to the loss for every image during training.

For the second MoI, the training data set is run through the model once to obtain a set of predicted labels for the images. A table or a dataframe (such as Figure S2) is created which includes the predicted probability that an image belongs to the “good porosity” class, the predicted probability that an image belongs to the “bad porosity” class, the predicted label (which is binary—0 for the “good porosity” class or 1 for the “bad porosity” class), the true (binary) label, and the normalized relative errors between the empirical maximum melt pool temperature and the simulated maximum melt pool temperature. For example, Figure S2 contains the physical data used in $L_{phy,1}$. Then, the model is trained. For each image it evaluates, if the predicted label does not match the actual label, the dataframe is used to identify the physical data corresponding to that image. These physical data are then incorporated into the loss function using one of the physics-informed custom loss functions. However, the standard categorical cross-entropy function is active if the predicted label and true label match. Through this process, this second MoI seeks to mimic the behavior of the standard categorical cross-entropy function, for which the loss increases whenever the true and predicted labels for an image do not match.

While the third MoI utilizes a table containing the same elements as the second, instead of targeting images for which the true and predicted label do not match, it only targets “bad” images that the model incorrectly labels as “good”. In other words, for each image the model evaluates, if the predicted label is “good”, but the true label is “bad”, the table identifies the physical data which correspond to that image. These physical data are then incorporated into one of the custom loss functions. However, in any other case, the standard categorical cross-entropy function is used. Through this process, the third MoI seeks to mimic the behavior of the standard categorical cross-entropy function while also shifting particular focus towards correctly identifying the images that contain the porosity defects.

## 4. Results

### 4.1. Training, Validation, Testing Splits

**Figure 4:**
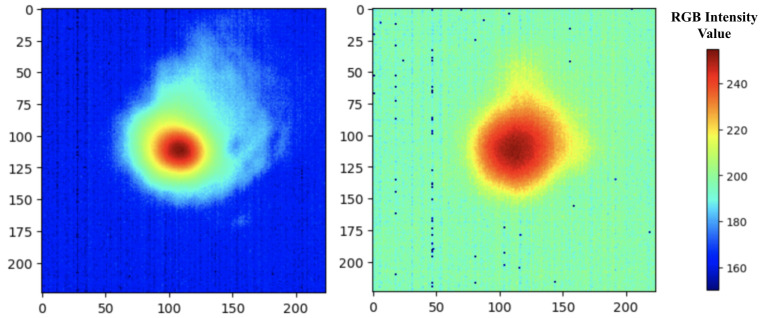
An example of a “good” pyrometer image (**left**) and a “bad” pyrometer image (**right**).

To properly train and assess the model, each image has a distinct porosity class for the model to predict. The labeling procedure described in Section 2.1 results in 1486 “good” labeled images, 71 “bad” labeled images, and 7 images that could not be labeled. Those unlabeled images are discarded from the analysis henceforth. Figure 4 provides an example of a “good” image as indicated by a spread of temperature distribution (color ranges from red to green) and a “bad” image indicated by a very hot temperature distribution (mostly red) for the melt pool. In Figure 4, the scales of the x-axis and y-axis refer to the pixel row and pixel column number, respectively. The legend refers to a subset of the 0–255 RGB intensity value color scale; the lowest value of this subset is 150 (corresponding to 0 ${}^{{}^{\circ}}$C) and the highest is 255 (corresponding to 2107 ${}^{{}^{\circ}}$C). The observed melt pool temperatures ranged from 0–2107 ${}^{{}^{\circ}}$C. Higher RGB intensity values correspond to higher melt pool temperatures. The CT scan helps to indicate the location of the part containing identified pores. These locations were matched to the melt pool image locations. While the CT scan provides the pore location by identifying pores, the melt pool images that match the pore locations reveal the temperature distribution that result in porous melt pools.

Training and assessing the performance of a CNN requires sorting images into a training set and a test set. In addition, a portion of the training set is used as a validation set to help carry out model selection by evaluating the accuracy of the model after each training epoch, while the test set is used to assess the performance of the model selected from the validation set on unseen data. The 1557 pyrometer images are therefore randomly sorted into a training set containing 1237 “good” and 59 “bad” images and a test set containing 249 “good” and 12 “bad” images. In the current state, bias during training will occur since there is an imbalanced data set (there are a lot more “good” images than “bad”). To fix this imbalance, the “bad” images from the training set are augmented to create additional “bad” images (as will be described in Section 4.2). The final training set contains 1237 “good” and 1237 “bad” images. A validation set consisting of 15% of the images in the training set (185 “good” and 185 “bad” images) is then randomly portioned out.

### 4.2. Data Pre-Processing and Augmentation

Each empirical melt pool CSV file is converted to a grayscale image using OpenCV, an open-source Python library for computer vision and machine learning. Each pixel within each image scales to a value between 0 and 255. Next, OpenCV’s minMaxLoc function locates the maximum temperature (denoted by the highest value pixel) for each melt pool (each grayscale image). The images are cropped into 224 × 224-pixel squares centered around the maximum temperature location. The crop allows OpenCV to tailor the images to the proper input size for the VGG16 CNN architecture and remove extraneous background pixels. The “jet” colormap is subsequently used to pseudocolor the images, converting them from grayscale to RGB format.

**Figure 5:**
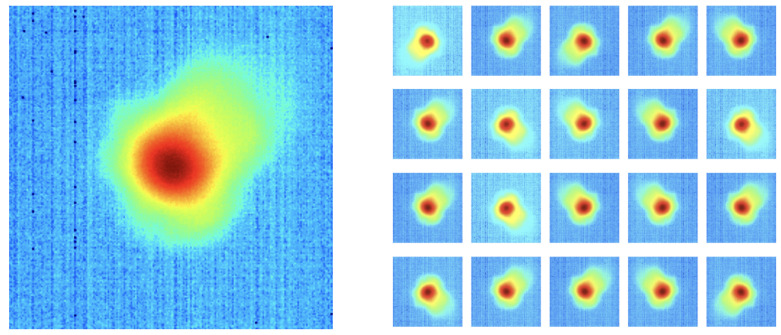
An example of a “bad” pyrometer image (**left**) and the 20 additional images created from it via data augmentation methods (**right**).

As is mentioned in Section 4.1, there are significantly more “good” images in the training data set (1237) than “bad” images (59). The “bad” images from the training set are augmented to create additional data to remedy this class imbalance and minimize bias during model training. For 57 of the 59 original “bad” images, 20 other images are created by randomly flipping the images horizontally, vertically, or both and altering the brightness with a random value in the range [0.7, 1.3]. For each of the two remaining original “bad” images, data augmentation creates 19 additional images using the aforementioned bootstrapping methods. This process results in 1237 different “bad” images, the same as the number of “good” images in the training data set. Figure 5 shows examples of these augmented images.

### 4.3. Results

Three metrics assess the performance of each version of the model: accuracy, weighted average precision, and weighted average recall. These metrics capture the effectiveness of the network in an intuitive fashion. In particular, a weighted average of both the precision and recall values is used to account for the class imbalance in the test set (which is made up of 95% “good” images and 5% “bad” images). Each metric uses the true porosity labels measured by micro-CT and the predicted porosity labels generated by the model as follows:

(6) $$Accuracy=\frac{TP+TN}{TP+FP+TN+FN}\times 100$$

(7) $$Precision\;\left(weighted\;average\right)=\left(B\times \frac{TP}{TP+FP}+G\times \frac{TN}{TN+FN}\right)\times 100$$

(8) $$Recall\;\left(weighted\;average\right)=\left(B\times \frac{TP}{TP+FN}+G\times \frac{TN}{TN+FP}\right)\times 100$$

where *B* and *G* are the percentages of the data set comprised of “bad” and “good” images, respectively. Note that the “bad” porosity class is positive, and the “good” porosity class is negative. Therefore, in Equations (6) to (8), TP denotes a true positive, or the number of correctly identified “bad” images. Similarly, TN denotes a true negative, or the number of correctly identified “good” images. Likewise, FP denotes a false positive, or the number of instances of the model predicting that the label of a “good” image is “bad”. FN denotes a false negative, or the number of instances of the model predicting that the label of a “bad” image is “good” [26]. Live plots keep track of the accuracy and loss of the model after each training epoch.

Let $PICNN_{i}$ (i=1,...,5) be the PICNN that uses $L_{phy,i}$ as the loss function. For each training session, the model trains for 100 epochs but is stopped early if the validation loss does not improve for 50 consecutive epochs. Furthermore, the learning rate is set to 0.001, the batch size is set to 16, and the optimizer used is the stochastic gradient descent method. This small learning rate and batch size are selected to optimize accuracy, as decreasing the learning rate and batch size are standard techniques for enhancing model performance. Google Colab, which uses a 12 GB NVIDIA Tesla K80 GPU, runs all training sessions. The best weights (based on lowest validation loss) from each training session generate predictions for the training and validation data sets.

First, $PICNN_{1}$ is trained via each of the three MoIs (see Section 3.3). In brief, the first MoI involves adding physical data to the loss for every image during training, the second MoI involves adding physical data to the loss only if the predicted label does not match the true label for a given image, and the third MoI involves adding physical data to the loss only if the predicted label is “good” but the true label is “bad” for a given image. Table 1 shows the accuracy, weighted average precision, and weighted average recall for both the training and validation data sets for $PICNN_{1}$ when trained via each MoI. Table 1 also shows the number of true positives, true negatives, false positives, and false negatives concerning the training and validation data sets for $PICNN_{1}$ when trained via each MoI.

One can observe from Table 1 that the accuracy in the validation set is highest with the first and third MoIs, though the accuracy does not vary much from one MoI to the other. Additionally, note that each of the three MoIs yields the same weighted average precision and weighted average recall values concerning the test data set. However, Table 1 does not deduce the main difference between the three MoIs: training time. The training session using the first MoI lasts approximately two hours, while the training sessions using the second and third MoIs last about thirteen and eleven hours, respectively. This discrepancy seems to result from the extra steps needed to traverse the dataframe (Figure S2) in the second and third MoIs. Thus, since the performance metrics for each of the three MoIs are similar, only the first MoI is used going forward to optimize both accuracy and training time.

Next, each version of PICNN uses the best MoI (i.e., the first MoI). A baseline deep learning-only model (see Section 3.1) is also trained using the regular Keras categorical cross-entropy loss function. Under these conditions, the baseline deep learning-only model achieves 98.86% accuracy, 99% precision (weighted average), and 99% recall (weighted average) on the training set. Table 2 shows the accuracy, weighted average precision, and weighted average recall for each version of the model (based on training set predictions). For $PICNN_{3}$ and $PICNN_{4}$, multiple values of $\lambda_{t}$ and $\lambda_{r}$, respectively, are tried. For $PICNN_{5}$, the values of $\lambda_{t}$ and $\lambda_{r}$ that yield the highest accuracy on the training set for $PICNN_{3}$ and $PICNN_{4}$ are chosen. Moreover, Table 3 shows the number of true positives, true negatives, false positives, and false negatives concerning the training data set for each version of the PICNN model.

Google Colab, which uses a 12GB NVIDIA Tesla K80 GPU, runs all predictions on the test set. Generating predictions on the test set takes approximately two to four minutes for each version of the model. The baseline deep learning-only model achieves 93.87% accuracy, 91% precision (weighted average), and 94% recall (weighted average) on the test set. Table 4 shows the accuracy, weighted average precision, and weighted average recall for each version of the model (based on test set predictions). For $PICNN_{3}$ and $PICNN_{4}$, multiple values of $\lambda_{t}$ and $\lambda_{r}$, respectively, are compared. For $PICNN_{5}$, the values of $\lambda_{t}$ and $\lambda_{r}$ that yielded the highest accuracy on the training set for $PICNN_{3}$ and $PICNN_{4}$ are chosen. Table 5 shows results on the test data set regarding the number of true positives, true negatives, false positives, and false negatives for each version of the PICNN model.

### 4.4. Discussion

The accuracy, weighted average precision, and weighted average recall values for the training data set for each version of PICNN are lower than for the baseline deep learning-only model. However, the discrepancy between the baseline and physics-informed models’ performance is smaller when evaluating the test set data. Notably, when tested on the test data set, the version of $PICNN_{3}$ in which $\lambda_{t}=0.5$, as well as the version of $PICNN_{4}$ in which $\lambda_{r}=0.5$ or 0.05, yielded a 1% increase in weighted average precision over the baseline deep learning-only model (with the other models yielding a weighted average precision equal to that of the baseline deep learning-only model). Furthermore, while the weighted average precision and recall values for the test data set are overall satisfactory, a closer look at Table 5, which shows the actual number of true positives, true negatives, false positives, and false negatives with regard to the test data set for each version of the model, shows a consistent issue across all versions: a low number of true positive predictions. The baseline model yielded 1028 true positives, 1052 true negatives, 0 false positives, and 24 false negatives when evaluating the training set. Therefore, the low number of true positive predictions from each version of the model when evaluating the test set could result from the model overfitting to the training set.

Furthermore, in terms of general patterns across the data, note that accuracy and recall seem to be inversely related to precision, especially for $PICNN_{4}$. For $PICNN_{3}$, as $\lambda_{t}$ decreases, accuracy and recall increase (to a point) and then begin to decrease again. The opposite is true for precision. For $PICNN_{4}$, as $\lambda_{r}$ decreases, accuracy and recall also decrease. Again, the opposite is true for precision.

While the described method of incorporating physics-informed constraints into the loss function of the model only resulted in a performance increase with respect to weighted average precision and not overall accuracy, this investigation has additional implications. This experiment poses a novel way to incorporate physical data into a CNN model in a way that integrates said data into the deep learning model architecture itself rather than appending additional reasoning onto the end of the process. Furthermore, it shows a way to incorporate both simulated physical data from an FEA model and empirical physical data into the model.

We note that our baseline deep learning-only model was not able to achieve the accuracy, precision, and recall of the PyroNet model on which it was based [26], which is due to differences in hyperparameter settings, data augmentation, and data partition. We also note that due to its magnitude, the improvement in weighted average precision between the baseline model and some versions of $PICNN_{3}$ and $PICNN_{4}$, respectively, could potentially be the result of different training instances. Further experiments are needed to determine the causes of performance differences between each of the models, which could then be used to further tune the model.

This experiment is focused on the choice of empirical physical data incorporated into the loss function. While the FEA model was used to calculate several additional variables, simulated melt pool length, simulated melt pool width, and simulated maximum melt pool temperature were chosen in conjunction with their corresponding empirical measures for simplicity. Future directions for this research could include an investigation of the impact of different and more complex physical data on the loss function. A comparison of how constraints informed only by the simulated physical data from the FEA and constraints informed only by the empirical physical data impact model performance could also be explored. Furthermore, this experiment focused on the objective function of the PICNN model. The addition of physics-informed constraints on the standard CNN hyperparameters (e.g., kernel size, padding, stride) and other changes to the overall architecture of the CNN may improve upon the results produced by the method described in this work and are potential directions for future research.

## 5. Conclusions

This work shows how incorporating both simulated and empirical physical LMD data into a deep learning model by means of altering the loss function impacts the accuracy, precision, and recall of said model as it attempts to predict porosity. While this physics-informed model cannot improve the predictive capabilities of the deep learning model in every respect, some versions can improve the precision of the baseline model. This improvement in precision is due to an increase in the number of true positive predictions, but comes at the expense of the model’s overall accuracy. Nonetheless, within the context of our experiment, precision is a crucial metric. This increase in precision indicates that some versions of the PICNN model have a higher true positive to total positive prediction ratio than the baseline model, and therefore have some advantage over the baseline deep learning-only model when it comes to identifying porosity. Additionally, while the second and third MoIs were not more effective at identifying porosity defects in this case study, they exhibited a novel approach to incorporating physical data into the CNN architecture via a custom loss function. More generally, they displayed that it is possible to utilize said methods to gain greater insight into and control over loss calculation when training a CNN. Overall, these results indicate that further research on this topic is a viable and worthwhile endeavor. Future directions for this work may include implementing further changes to the loss function, the addition of physics-informed constraints on the standard hyperparameters, and other changes to the architecture of the CNN as a whole.

## Figures and Tables

**Table 1:** Performance metrics for $PICNN_{1}$ with respect to the training and validation data sets when the model is trained via each MoI.

MoI	Set	Accuracy (%)	Precision (%)	Recall (%)	TP	TN	FP	FN
			(Weighted Avg.)	(Weighted Avg.)				
1	Train	82.22	84	82	757	973	79	295
	Val	92.70	93	93	174	169	16	11
2	Train	84.07	85	84	790	979	73	262
	Val	92.43	93	93	174	168	17	11
3	Train	84.51	86	85	792	986	66	260
	Val	92.70	93	93	174	169	16	11

**Table 2:** Performance metrics for the baseline deep learning-only model and each version of the physics-informed model when tested on the training data set.

Model	$\lambda_{t}$	$\lambda_{r}$	Accuracy (%)	Precision (%)	Recall (%)
				(Weighted Avg.)	(Weighted Avg.)
Deep Learning-Only	-	-	98.86	99	99
$PICNN_{1}$	-	-	82.22	84	82
$PICNN_{2}$	-	-	80.42	82	80
$PICNN_{3}$	1	-	83.41	84	83
	0.5	-	85.12	87	85
	0.05	-	84.13	85	84
$PICNN_{4}$	-	1	82.32	83	82
	-	0.5	81.42	81	81
	-	0.05	79.56	80	80
$PICNN_{5}$	0.5	1	82.13	85	82

**Table 3:** The number of true positive (TP), true negative (TN), false positive (FP), and false negative (FN) predictions for the baseline deep learning-only model and each version of the physics-informed model when tested on the training data set.

Model	$\lambda_{t}$	$\lambda_{r}$	TP	TN	FP	FN
Deep Learning-Only	-	-	1028	1052	0	24
$PICNN_{1}$	-	-	757	973	79	295
$PICNN_{2}$	-	-	714	978	74	338
$PICNN_{3}$	1	-	793	962	90	259
	0.5	-	775	1016	36	277
	0.05	-	783	987	65	269
$PICNN_{4}$	-	1	778	954	96	274
	-	0.5	851	862	190	201
	-	0.05	884	790	262	168
$PICNN_{5}$	0.5	1	716	1012	40	336

**Table 4:** Performance metrics for the baseline deep learning-only model and each version of the physics-informed model when tested on the test data set.

Model	$\lambda_{t}$	$\lambda_{r}$	Accuracy (%)	Precision (%)	Recall (%)
				(Weighted Avg.)	(Weighted Avg.)
Deep Learning-Only	-	-	93.87	91	94
$PICNN_{1}$	-	-	88.89	91	89
$PICNN_{2}$	-	-	88.51	91	89
$PICNN_{3}$	1	-	87.36	91	87
	0.5	-	91.57	92	92
	0.05	-	89.27	91	89
$PICNN_{4}$	-	1	86.97	91	87
	-	0.5	79.31	92	79
	-	0.05	72.80	92	73
$PICNN_{5}$	0.5	1	91.57	91	92

**Table 5:** The number of true positive (TP), true negative (TN), false positive (FP), and false negative (FN) predictions for the baseline deep learning-only model and each version of the physics-informed model when tested on the test data set.

Model	$\lambda_{t}$	$\lambda_{r}$	TP	TN	FP	FN
Deep Learning-Only	-	-	0	245	4	12
$PICNN_{1}$	-	-	1	231	18	11
$PICNN_{2}$	-	-	1	230	19	11
$PICNN_{3}$	1	-	1	227	22	11
	0.5	-	1	238	11	11
	0.05	-	1	232	17	11
$PICNN_{4}$	-	1	1	226	23	11
	-	0.5	3	204	45	9
	-	0.05	4	186	63	8
$PICNN_{5}$	0.5	1	0	239	10	12

## Supplementary Materials

The following are available online at https://www.mdpi.com/article/10.3390/s1010000/s1, Figure S1: Hyperparameters of the PyroNet, Figure S2: An example of a table in the form of a dataframe that can be used to train the model via the second or third MoI. In the text, tempErrNorm is denoted ${\hat{\epsilon }}_{t_{i}}$.
