# Supplementary figures and images for: A Physics-Informed Convolutional Neural Network with Custom Loss Functions for Porosity Prediction in Laser Metal Deposition
Source: Sensors (Basel). 2022 Jan 10;22(2):494. doi: 10.3390/s22020494 (PMC8779806; doi:10.3390/s22020494)

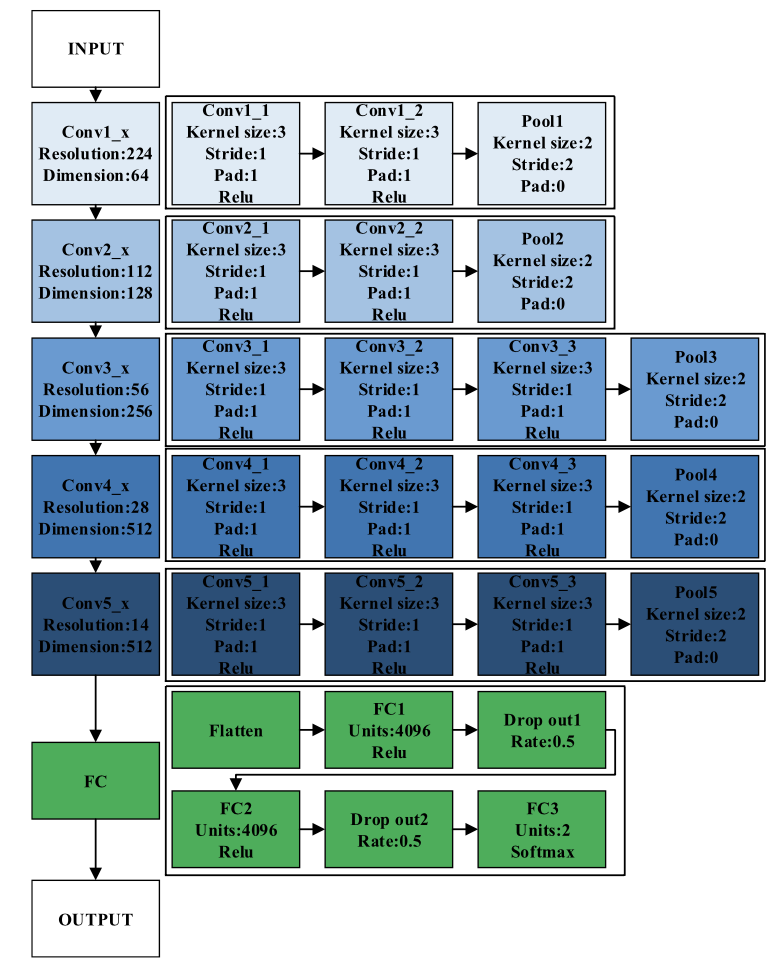

Supplement: Supplementary file 1 [file sensors-22-00494-s001.zip › Figure S1.Hyperparameters.png]

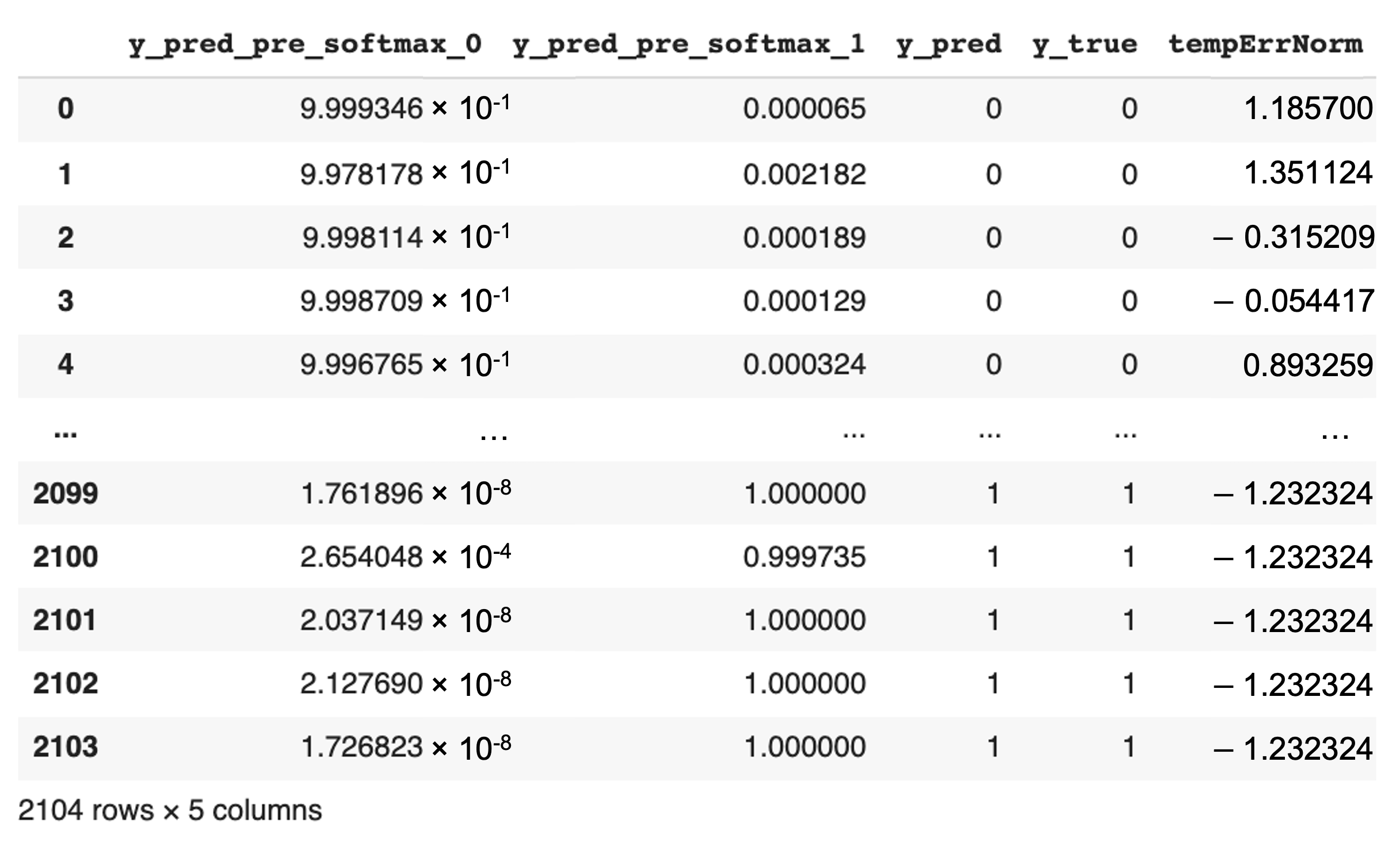

Supplement: Supplementary file 1 [file sensors-22-00494-s001.zip › Figure S2. DataFrame2.png]
